# Impact of obesity on intensive care outcomes in patients with COVID-19 in Sweden—A cohort study

**DOI:** 10.1371/journal.pone.0257891

**Published:** 2021-10-13

**Authors:** Lovisa Sjögren, Erik Stenberg, Meena Thuccani, Jari Martikainen, Christian Rylander, Ville Wallenius, Torsten Olbers, Jenny M. Kindblom

**Affiliations:** 1 Department of Internal Medicine and Clinical Nutrition, Institute of Medicine, Sahlgrenska Academy at University of Gothenburg, Gothenburg, Sweden; 2 Department of Pediatrics, Institute of Clinical Sciences, The Sahlgrenska Academy, University of Gothenburg, Gothenburg, Sweden; 3 Department of Pediatrics, Hallands Hospital Halmstad, Halmstad, Sweden; 4 Department of Surgery, Faculty of Medicine and Health, Örebro University, Örebro, Sweden; 5 Department of Anaesthesiology and Intensive Care Medicine, Institute of Clinical Sciences, Sahlgrenska Academy, University of Gothenburg, Gothenburg, Sweden; 6 Bioinformatics Core Facility, The Sahlgrenska Academy, University of Gothenburg, Gothenburg, Sweden; 7 Department of Surgery, Institute of Clinical Sciences, Sahlgrenska Academy, University of Gothenburg, Gothenburg, Sweden; 8 Dept of Biomedical and Clinical Sciences, Linköping University, Linköping, Sweden; 9 Pediatric Clinical Research Center, Sahlgrenska University Hospital, Region Västra Götaland, Gothenburg, Sweden; Heidelberg University Hospital, GERMANY

## Abstract

**Background:**

Previous studies have shown that a high body mass index (BMI) is a risk factor for severe COVID-19. The aim of the present study was to assess whether a high BMI affects the risk of death or prolonged length of stay (LOS) in patients with COVID-19 during intensive care in Sweden.

**Methods and findings:**

In this observational, register-based study, we included patients with COVID-19 from the Swedish Intensive Care Registry admitted to intensive care units (ICUs) in Sweden. Outcomes assessed were death during intensive care and ICU LOS ≥14 days. We used logistic regression models to evaluate the association (odds ratio [OR] and 95% confidence interval [CI]) between BMI and the outcomes. Valid weight and height information could be retrieved in 1,649 patients (1,227 (74.4%) males) with COVID-19. We found a significant association between BMI and the risk of the composite outcome death or LOS ≥14 days in survivors (OR per standard deviation [SD] increase 1.30, 95%CI 1.16–1.44, adjusted for sex, age and comorbidities), and this association remained after further adjustment for severity of illness (simplified acute physiology score; SAPS3) at ICU admission (OR 1.30 per SD, 95%CI 1.17–1.45). Individuals with a BMI ≥ 35 kg/m^2^ had a doubled risk of the composite outcome. A high BMI was also associated with death during intensive care and a prolonged LOS in survivors assessed as separate outcomes.

The main limitations were the restriction to the first wave of the pandemic, and the lack of information on socioeconomic status as well as smoking.

**Conclusions:**

In this large cohort of Swedish ICU patients with COVID-19, a high BMI was associated with increasing risk of death and prolonged length of stay in the ICU. Based on our findings, we suggest that individuals with obesity should be more closely monitored when hospitalized for COVID-19.

## Introduction

Coronavirus disease 2019 (COVID-19), caused by severe acute respiratory syndrome coronavirus 2 (SARS-CoV-2) infection, includes a wide range of clinical presentations; from asymptomatic to severe pneumonia with acute respiratory injury (ARI) or acute respiratory distress syndrome (ARDS) in about 20% of patients seeking medical care due to COVID-19 [1–6]. The COVID-19 pandemic with rapidly increased demand for healthcare in hospitals and intensive care units (ICU) has placed an unprecedented strain on health care systems globally. The cumulative number of deaths due to SARS Cov-2 was 3,916,771 according to the World Health Organization report and the number of known cases was 180,492,131 cases [7]. Sweden was hit hard by the pandemic with 13.880 reported deaths by June 2021 [8].

Old age, male sex, diabetes, hypertension, cardiac disease as well as liver and kidney disease have been identified as risk factors for severe COVID-19 disease [9–12]. Obesity, a chronic and severe disease [13–15] that increases the risk of comorbidities such as type 2 diabetes and hypertension, was also identified as a clinical risk factor for severe COVID-19 in the early stages of the pandemic [16–19]. Since then, several observational studies have consistently found obesity to be associated with increased risk of more severe disease and hospitalization. A well-powered study in the UK demonstrated an association between BMI and the risk of hospitalization and death due to COVID-19 [20], and a case-control study using Swedish registers, found obesity to be an independent risk factor for severe disease [21]. A large multinational prospective study investigating ICU outcomes found that 90 day mortality was higher in patients with obesity [22]. Moreover, a recent Mendelian Randomization study concluded that a high BMI is causally related to COVID-19 susceptibility and severity [23]. In addition, obesity has been shown to increase the risk of prolonged hospitalization, critical illness, and need for mechanical ventilation in association with other respiratory infectious diseases such as influenza and pneumonia [24–28]. A prolonged length of stay (LOS) in intensive care reflects a more severe disease with a need for long rehabilitation [29–31].

Thus, there is evidence to support a link between obesity and severe COVID-19, and between obesity and intensive care outcomes in other respiratory infections. However, less is known about the association between elevated BMI and death and LOS during intensive care in patients with COVID-19.

We hypothesized that obesity has severe impact on intensive care outcomes, and aimed to evaluate the association between obesity and death and prolonged LOS in a large cohort with COVID-19 treated in Swedish ICUs during the first wave of the pandemic.

## Materials and methods

In this national observational study, we retrieved data from the Swedish Intensive Care Registry (SIR), medical records and the Swedish Passport Register on patients with COVID-19 admitted to ICUs in Sweden during the first wave of the COVID-19 pandemic. The SIR is a national quality registry that collects data on intensive care patients in Sweden with a reported 100% coverage for patients treated for severe COVID-19 (www.icuregswe.org). The registry contains data on diagnoses, interventions and inpatient care from the Swedish intensive care units. The Swedish Passport Register includes self-reported heights for all individuals holding a passport in Sweden since 1991.

The inclusion criteria for the present study were intensive care treatment during the study period March 6 2020, until August 23 2020, a diagnosis of COVID-19, age ≥18 years, and data available on height and recent weight. Exclusion criteria were age <18 years, pregnancy, or missing data on height or missing or outdated weight.

The study was approved by the Swedish Ethical Review Authority (Dnr 2020–03051).

### Anthropomentric data and covariates

**Fig 1:**
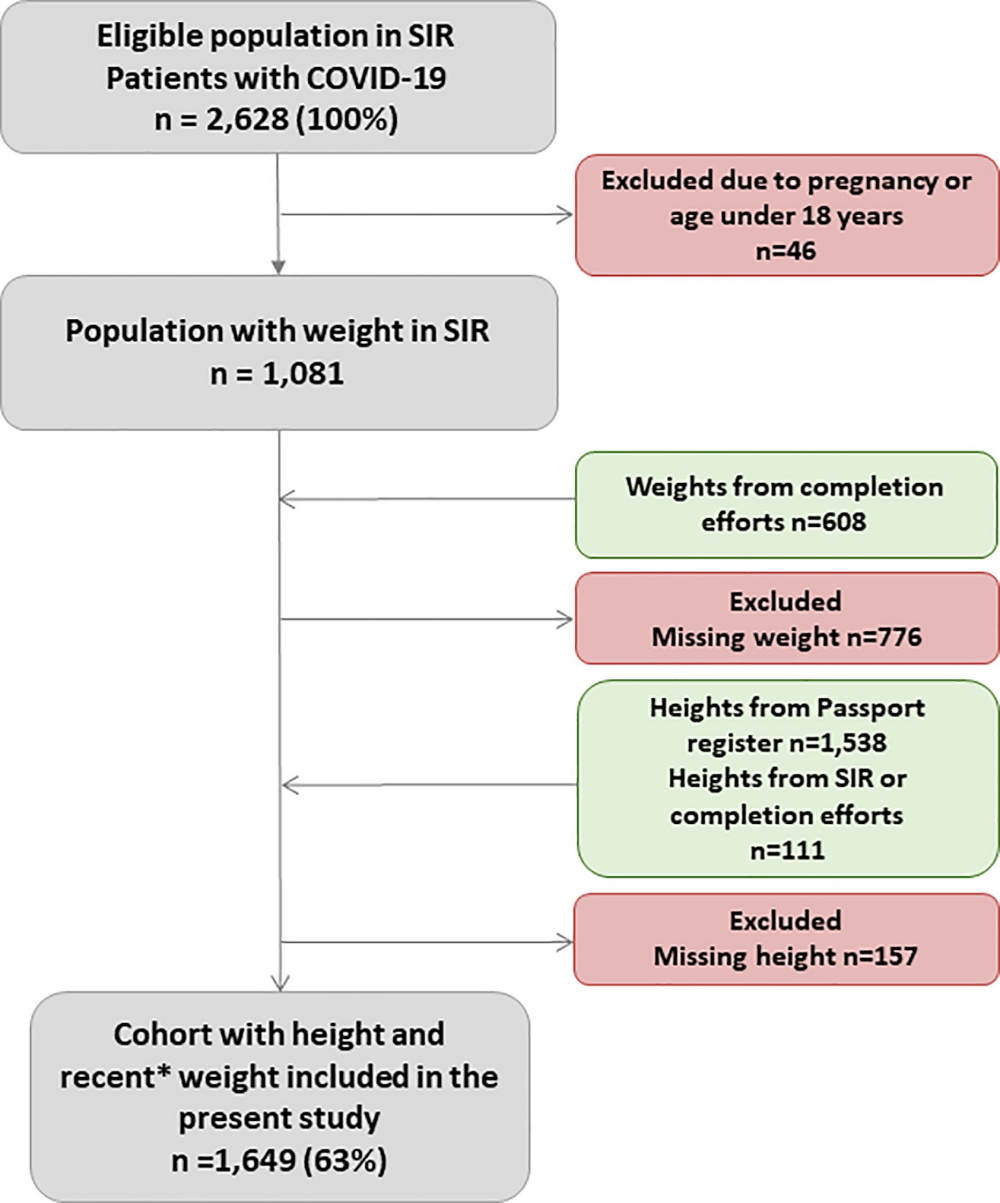
Flow chart of inclusion in the present study cohort. SIR = Swedish Intensive Care Registry.

Among the eligible 2,628 patients, 1,081 had a weight registered in SIR at ICU admission. The ICUs were subsequently approached by telephone and mail and asked to complete missing weight and height data. About half of the sites contributed data. To ensure that current weights were used in the study, we only included recent measurements (not more than three months prior to ICU admission) or measurements at admission to ICU. In addition, height data was retrieved from the Swedish Passport register (n = 1,538). For individuals missing in the Swedish Passport Register, we used SIR data or completed data. The inclusions and exclusions are presented in Fig 1.

We calculated BMI as weight in kilograms divided by squared height in meters (kg/m^2^) and defined overweight as ≥25 kg/m^2^, class 1 obesity as ≥30 kg/m^2^, class 2 obesity as ≥35 kg/m^2^, and class 3 obesity as ≥40 kg/m^2^, respectively [32]. The group with a BMI between 18 and 25 kg/m^2^ has been used as the reference throughout the paper, referred to as the reference group.

The comorbidities were defined according to the International classification of disease (ICD) 10 (cardiovascular disease I00-I99; hypertension I10-I15; diabetes E10-E14; kidney disease N18; liver disease K70-K77).

The simplified acute physiology score (SAPS) 3 was developed as an instrument to balance the performance of ICUs against the severity of their actual case-mix [33] and represents an assessment of severity of illness at admission to ICU. Among other data, it includes chronic diseases and acute physiological derangements [33]. All patients with data available on type of ventilation (n = 1,180) had received mechanical ventilation.

### SARS-CoV2 diagnostics

All patients were diagnosed with COVID-19 through testing for SARS-CoV-2 using real-time reverse transcriptase-polymerase chain reaction (PCR) assays or through clinical diagnosis. We defined primary COVID-19 as any of the following ICD10 diagnoses: U071, U072, J09-J18, J80 or J96 as a main diagnosis.

### Outcomes

To investigate the importance of high BMI for outcomes reflecting severe COVID-19 during intensive care in both survivors and non-survivors, we evaluated the association between BMI and the composite of death during intensive care and LOS in survivors ≥14 days. We also evaluated the impact of BMI on these outcomes separately. We used LOS in survivors exceeding 14 days as a marker of more severe disease based on a previous study that reported a median length of stay of 14 days for patients treated in the ICU for COVID-19 [34]. As an exploratory outcome, we also evaluated pulmonary embolism (defined as ICD10 code I26).

### Statistical analyses

Descriptive characteristics are presented as mean (standard deviation [SD]) for continuous variables and n(%) for dichotomous variables. We compared means using student´s t test and frequencies using chi-square test. We tested the number of men vs women using a binomial test. Associations were evaluated using logistic regression models. The models include the outcome as dependent variable, and the exposure (BMI as a continuous or dichotomous variable), and covariates as independent variables, and are presented as odds ratios (ORs) with 95% confidence intervals (CI). The models were adjusted for covariates sex, age, presence of any (one or several) of the comorbidities (including cardiovascular disease, hypertension, diabetes, kidney and liver disease) and SAPS3 at ICU admission, as specified. We used linear regression with similar modelling for the continuous outcome length of stay. Possible interactions were evaluated by addition of interaction terms in the logistic regression models. Possible non-linear associations were evaluated by inclusion of a quadratic term for BMI. A p-value <0.05 for was interpreted as statistically significant.

## Results

In this nationwide register study, 2,628 eligible patients treated in ICUs during the study period, had a PCR verified or clinical COVID-19 diagnosis (Fig 1). 1,649 (63%) fulfilled the inclusion criteria (Fig 1). The included study cohort had a mean age of 60.1 years (SD 13.0) and were predominantly male (74%, p<0.001). A majority (95.6%) of the study cohort had PCR verified SARS-CoV-2 infection. Clinical characteristics are listed in Table 1 and S1 Table.

**Table 1:** Cohort description.

	N	Total cohort	Males	Females
Sex n(%)	1,649	1,649	1,227 (74.4)	422 (25.6)
Age (years)	1,649	60.1 (13.0)	59.9 (12.8)	60.6 (13.6)
BMI kg/m^2^	1,649	29.5 (6.1)	29.2 (5.6)	30.4 (7.4)
Weight (kg)	1,649	89.2 (20.5)	91.7 (19.8)	81.7 (20.9)
Height (cm)	1,649	173.7 (9.6)	177.2 (7.7)	163.8 (7.2)
Lab-verified COVID-19	1,649	1,577 (95.6)	1,175 (95.8)	402 (95.3)
Length of stay (days)	1,649	14.6 (13.7)	15.3(13.9)	12.6 (12.9)
Length of stay ≥14 days n(%)	1,649	661 (40.1)	524(42.7)	137 (32.5)
Death during stay at ICU (n(%)	1,649	333(20.2)	260(21.2)	73 (17.3)
Pulmonary embolism n(%)	1,649	71(4.3)	53(4.3)	18(4.3)
*Comorbidities*				
CVD n(%)	1,649	544 (33.0)	419 (34.1)	125 (29.6)
Hypertension	1,649	317 (19.2)	238 (19.4)	79 (18.7)
Diabetes n(%)	1,649	193 (11.7)	142 (11.6)	51 (12.1)
Kidney disease n(%)	1,649	38 (2.3)	32 (2.6)	6 (1.4)
Liver disease n(%)	1,649	21 (1.3)	12 (1.0)	9 (2.1)
*ICU scores/variables at admittance*				
SAPS3	1,648	53.9 (10.7)	53.5 (10.7)	55.1 (10.7)
PaO_2_ (kPa)	1,438	10.5 (8.1)	10.5 (8.1)	10.5 (7.9)
FiO_2_ (%)	1,097	70.0 (21.0)	69.3 (21.1)	72.1 (20.4)
SOFA	555	3.92 (3.4)	3.8 (3.3)	4.3 (3.5)

A cohort of 1,649 Swedish men and women treated at intensive care units during the first wave of the corona virus pandemic 2020. The data is presented as mean (SD) for continuous variables and n(%) for dichotomous variables. SD = standard deviation, BMI = body mass index, CVD = cardiovascular disease, SAPS = simplified acute physiology score, PaO_2_ = partial pressure of oxygen, FiO_2_ = fraction inhaled oxygen, SOFA = sequential organ failure assessment score.

P<0.001 men vs women.

### Overweight and obesity and the association with the composite outcome

A majority of the study cohort had a high BMI; 78.3% were overweight or had obesity (≥25kg/m^2^), 39.4% had obesity (≥30kg/m^2^) and 14.9% had class 2 or 3 obesity (≥35kg/m^2^). There were significantly more women than men with a BMI above 30 kg/m^2^ (p<0.001) (S1 Table). The groups with overweight or different classes of obesity were significantly younger than the reference group, and had lower SAPS3 at ICU admission compared to the reference group, but only differed marginally regarding comorbidities (Table 2 and S1 Table). The number of underweight individuals (≤18.0kg/m^2^) were too few to be analyzed as a separate group (n = 15).

**Table 2:** Cohort characteristics across BMI strata.

	BMI categories			
	Reference group	Overweight	Obesity 1	Obesity 2 and 3
	N = 343	N = 641	N = 404	N = 246
**BMI (kg/m** ^**2**^ **)**	22.9(1.7)	27.4(1.4)***	32.1(1.4)***	40.4(5.3)***
**Age (years)**	63.1(13.1)	61.6(12.4) NS	58.1(12.1)***	54.613.6)***
**Length of stay (days)**	13.6(13.7)	14.8(12.9) NS	14.9(14.4) NS	15.1(12.8) *
**SAPS3 at admission**	56.3(11.6)	54.0(10.3)**	52.6(10.2) ***	52.3(10.7)***
**CVD n(%)**	108 (31.5)	225 (35.1)NS	132 (32.7) NS	75 (30.5) NS
**Hypertension n(%)**	55 (16.0)	138 (21.5)*	75 (18.6) NS	49(19.9) NS
**Diabetes n(%)**	31(9.0)	74 (11.5) NS	54 (13.4) NS	32 (13.0) NS
**Kidney disease n(%)**	9 (2.6)	15 (4.4) NS	10 (2.5) NS	4 (1.6) NS
**Liver disease n(%)**	6 (1.7)	9 (1.4) NS	3 (0.7) NS	3 (1.2) NS

Characteristics according to BMI strata presented as mean (SD) or n(%). Student´s t test (for continuous variables) and Chi square test (for dichotomous variables) vs the reference group (≥18, <25 kg/m^2^).

*** p<0.001

** p<0.01

*p<0.05, NS = not significant.

Reference group = ≥18, <25 kg/m^2^; Overweight = ≥25, <30 kg/m^2^; Obesity 1 = ≥30, <35 kg/m^2^; Obesity 2 and 3 = ≥35 kg/m^2^.

BMI = body mass index, SAPS = simplified acute physiology score, CVD = cardiovascular disease.

We found a significant association between BMI as a continuous variable and the composite outcome death during intensive care or LOS in survivors ≥14 days (OR per SD increase: 1.30 95%CI 1.16–1.44 adjusted for age and sex), and this association remained also after adjusting for presence of any (one or several) of the comorbidities cardiovascular disease, hypertension, diabetes mellitus, liver or kidney disease (Table 3, Panel A). In the same model, male sex (OR 1.69 males vs females, 95%CI 1.34–2.13), high age (OR 1.03 per year 95%CI 1.02–1.03) and presence of any of the comorbidities (OR 1.95, 95%CI 1.58–2.4) was also significantly associated with the composite outcome.

**Table 3:** BMI and BMI categories and the risk of composite outcome, death during ICU or ICU stay over 14 days.

	N (cases)	Unadjusted model	Adjusted model 1	Adjusted model 2	Adjusted model 3
		***Panel A*: *Composite outcome***			
		**OR per SD (95%SD)**	**OR per SD (95%SD)**	**OR per SD (95%SD)**	**OR per SD (95%SD)**
**BMI continuous**	1,649 (848)	1.14(1.03–1.26)	1.29(1.16–1.43)	1.30(1.16–1.44)	1.30(1.17–1.45)
**BMI category**		**OR(95% CI)**	**OR(95% CI)**	**OR(95% CI)**	**OR(95% CI)**
**Reference group**	343 (166)	Reference	Reference	Reference	Reference
**Overweight**	641 (325)	1.10(0.84–1.43)	1.12(0.85–1.46)	1.10(0.83–1.44)	1.14(0.87–1.51)
**Obesity 1**	404 (210)	1.15(0.87–1.54)	1.36(1.00–1.84)	1.35(0.99–1.83)	1.40(1.03–1.92)
**Obesity 2 and 3**	246 (142)	1.46(1.05–2.02)	2.00(1.39–2.87)	1.97(1.36–2.85)	2.02(1.39–2.94)
		***Panel B*: *Death during intensive care***			
		**OR per SD (95%SD)**	**OR per SD (95%SD)**	**OR per SD (95%SD)**	**OR per SD (95%SD)**
**BMI continuous**	1,649 (333)	0.97(0.86–1.10)	1.20(1.05–1.37)	1.20(1.05–1.37)	1.19(1.04–1.36)
**BMI category**		**OR(95% CI)**	**OR(95% CI)**	**OR(95% CI)**	**OR(95% CI)**
**Reference group**	343 (69)	Reference	Reference	Reference	Reference
**Overweight**	641 (132)	1.03(0.74–1.43)	1.12(0.80–1.57)	1.10(0.79–1.55)	1.23(0.87–1.75)
**Obesity 1**	404 (83)	1.03(0.72–1.47)	1.49(1.01–2.20)	1.48(1.00–2.18)	1.54(1.04–2.30)
**Obesity 2 and 3**	246 (45)	0.89(0.59–1.35)	1.41(0.89–2.23)	1.37(0.86–2.17)	1.43(0.89–2.30)
		***Panel C*: *LOS ≥14 days in survivors***			
		**OR per SD (95%SD)**	**OR per SD (95%SD)**	**OR per SD (95%SD)**	**OR per SD (95%SD)**
**BMI continuous**	1,316 (515)	1.21(1.08–1.35)	1.29(1.14–1.45)	1.30(1.16–1.47)	1.31(1.16–1.48)
**BMI category**		**OR(95% CI)**	**OR(95% CI)**	**OR(95% CI)**	**OR(95% CI)**
**Reference group**	274 (97)	Reference	Reference	Reference	Reference
**Overweight**	509 (193)	1.11(0.82–1.51)	1.09(0.80–1.49)	1.08(0.79–1.47)	1.09(0.79–1.49)
**Obesity 1**	321 (127)	1.19(0.86–1.67)	1.25(0.89–1.77)	1.26(0.88–1.79)	1.29(0.91–1.85)
**Obesity 2 and 3**	201 (97)	1.70(1.17–2.47)	2.06(1.38–3.08)	2.08(1.39–3.12)	2.11(1.41–3.18)

Odds ratios (95% confidence intervals) for the composite outcome, death during intensive care or length of stay (LOS) at intensive care unit over 14 days, was calculated using logistic regression models. Adjusted model 1 is adjusted for age and sex, adjusted model 2 is adjusted for age, sex and comorbidities (presence of any of cardiovascular disease, hypertension, diabetes mellitus, liver- or kidney disease, n = 615), and adjusted model 3 is adjusted for age, sex, comorbidities and SAPS (simplified acute physiology score) 3. The reference group refers to individuals with BMI ≥18, <25 kg/m^2^.

Panel A: Total n = 1,649; composite outcome n = 848 (death during intensive care n = 333; LOS over 14 days n = 515).

Panel B: Total n 1,649, death during stay at ICU, n = 333.

Panel C: Total n = 1,311; LOS over 14 days in survivors, n = 515.

BMI = body mass index, OR = odds ratio, CI = confidence interval, LOS = length of stay.

BMI was only marginally associated with SAPS3 at ICU admission (variance explained r^2^ = 1.4%). To determine if SAPS3 at ICU admission still might be a partial mediator of the association between BMI and the composite outcome, we compared the logistic regression models with and without inclusion of SAPS3 as a covariate, revealing essentially unchanged point estimates (ORs 1.29 and 1.30, Table 3, Panel A). These findings indicate that the association between BMI and the composite outcome is not mediated via severity of illness as assessed by SAPS3 at ICU admission.

The risk of the composite outcome was significantly increased for class 1 obesity in the fully adjusted model (adjusted for age, sex, comorbidities and SAPS3) and doubled for class 2 and 3 obesity together (BMI≥ 35 kg/m^2^) in all the adjusted models, compared with the reference group (Table 3, Panel A). The test for non-linearity was non-significant (p for quadratic term for BMI>0.05), and no significant interactions between BMI and the covariates were detected.

When the analyses were re-performed including only individuals 55 years of age or less, the same pattern was seen (S2 Table).

### Associations between obesity and death during intensive care

BMI as a continuous variable was associated with the risk of death during the ICU stay (OR per SD increase 1.20, 95% CI 1.05–1.37, adjusted for age and sex). Importantly, this association remained also after adjustment for comorbidities and SAPS3 at ICU admission (Table 3, Panel B). We further observed a significant association between class 1 obesity, compared with the reference group, and the risk of death during intensive care, but for class 2 and 3 obesity the association did not reach statistical significance (Table 3, Panel B).

### Association between obesity and length of stay in survivors

Individuals with class 2 and 3 obesity had significantly longer LOS than the reference group (Table 2). We evaluated the association between BMI and the risk of LOS ≥14 days in survivors and found that BMI is a determinant of prolonged LOS in all models evaluated (Table 3 Panel C). Class 2 and 3 obesity together was associated with a doubled risk of LOS ≥14 days (OR 2.11 95% CI 1.41–3.18; Panel C, fully adjusted model 3) compared with the reference group. One SD higher BMI was associated with 1.35 days longer intensive care among survivors (95% CI 0.58–2.11) in a linear regression model adjusted for age, sex, comorbidities and SAPS3 at ICU admission.

### Exploratory and sensitivity analyses

In a less powered exploratory analysis, we evaluated the association between BMI and the risk of pulmonary embolism. Only 71 patients had a registered pulmonary embolism during the ICU stay. No significant association was seen between class 2 or 3 obesity and the risk of pulmonary embolism (OR 0.63, 95%CI 0.25–1.56), adjusted for age, sex, comorbidities and SAPS3 at ICU admission, compared with the reference group.

To test the robustness of the observed associations we also performed sensitivity analyses excluding individuals who did not have COVID-19 as primary diagnosis (n = 97). These analyses revealed materially similar results (S3 Table).

## Discussion

In this register-based study, we present detailed and recent BMI characteristics and associations with intensive care outcomes for a large cohort with COVID-19 in Swedish ICUs during the first wave of the COVID-19 pandemic. We demonstrate a significant association between high BMI and increased risk of the composite outcome death and prolonged LOS in patients with COVID-19 after adjusting for age, sex and comorbidities. Importantly, this association was not mediated via severity of illness at ICU admission. Individuals with a BMI > 35 kg/m^2^ had a doubled risk of death or prolonged intensive care. A high BMI was also associated with death during intensive care and a prolonged LOS in survivors assessed as separate outcomes.

Previous studies, both observational [11, 18, 20, 21, 35, 36] and studies using the Mendelian randomization approach [23], have consistently found higher susceptibility and severity of the COVID-19 disease course in individuals with obesity. A UK study including almost 7 million individuals concluded increased risk of hospitalization and death due to COVID-19 in individuals with obesity, but only a small sample of the included patients had a recent BMI measurement (i.e. within the past year) [20]. Another study that included over 17 million adults observed increased risk of COVID-19 related death with increasing obesity [11]. Of note, the study accepted weight measurements as old as 10 years before inclusion in the study and therefore, associations between a recent BMI and mortality could not be evaluated [11]. The COVID-ICU group found severe obesity to be a predictor of early mortality before 14 days, but did not report the association between BMI and death specifically during the ICU stay, or length of stay in the ICU among survivors [22]. Furthermore, one previous Swedish study [21] found an increased risk of severe COVID-19 in patients with obesity with the most pronounced excess risk in individuals younger than 56 years of age. The study, lacking actual BMI data, defined obesity based on the presence of the diagnosis E66 during the last 15 years in the National Patient Register. As the ICD code E66 in the Swedish National Patient Register does not reflect the true prevalence of obesity in Sweden (37), the association between BMI (as a continuous variable) and outcomes, or the separate associations for overweight and different classes of obesity, could not be evaluated in that study [21]. In the present study, using up-to-date BMI data, we found that a high BMI was associated with increased risk of death and prolonged intensive care in patients with severe COVID-19 after adjustment of age, sex and comorbidities. BMI was significantly associated with the composite outcome after further adjustment for SAPS3, indicating that the associations observed were not mediated via severity of illness as assessed by SAPS3 at ICU admission. By defining a composite outcome including death during intensive care and LOS over 14 days in survivors, we capture more severe illness both in survivors and non-survivors. The results from the present study establish class 2 and 3 obesity (BMI over 35 kg/m^2^) as a risk factor for the composite outcome death during intensive care and prolonged intensive care in patients with COVID-19. Moreover, given the findings of increased impact of obesity in younger individuals [21], we performed analyses restricted to patients younger than 55 years. We found materially similar associations in individuals 55 years or younger as in the complete cohort including individuals of all ages above 18 years.

We observed a significant association between BMI and the risk of death during intensive care, and with the risk of a prolonged LOS, evaluated as separate outcomes. Importantly, we found increased risk of death in individuals with class 1 obesity (≥30 kg/m^2^). For class 2 and 3 however, the association did not reach statistical significance, supposedly due to insufficient power. A prolonged LOS reflects a more severe course of disease and is associated with greater functional impairment in survivors [29–31]. We found that individuals with class 2 or 3 obesity had an almost two-fold risk of ICU LOS over 14 days, compared to patients without overweight or obesity. In a large prospective study, 94% of all participating ICUs reported that the COVID-19 pandemic led to a need of extended number of ICU beds [22]. Thus, a prolonged LOS is an important factor not only for the individual patient but also as an important factor for health care, putting excess strain on ICUs during the COVID-19 outbreak.

The mechanisms behind a more severe COVID-19 in patients with obesity are not fully known but may include impairments in the adaptive immune response [37], cardiometabolic and thrombotic derangements [38], and alterations in lung function [39]. However, the associations observed in the present study were essentially unaltered after adjustment for both comorbidities, including cardiovascular disease, hypertension, diabetes, liver and kidney disease, and severity of illness assessed as SAPS3 at ICU admission. Notably, SAPS3, including chronic diseases and acute physiological derangements but not body weight or BMI, showed only minor differences with little clinical importance between the different overweight and obesity strata, compared with the reference group.

In the present cohort including patients with COVID-19 in intensive care in Sweden, 39% of the included individuals had a BMI over 30 kg/m^2^. Our findings confirm an overrepresentation of obesity in the study cohort, compared to the background population [35] and the population in intensive care [14, 37], as previously described [11, 18, 20–22, 36]. We also confirmed a large overrepresentation of men in the included cohort, approximately three times as many men as women [22].

In contrast to previous studies reporting thromboembolic complications in 17% of patients with severe COVID-19 admitted to the ICU [22], the incidence of thromboembolism was only 4%. This discrepancy with other studies is likely explained by underreporting in the present study.

The strengths of the present study include the large population-based cohort with detailed, current BMI data. Moreover, the study population reflects Sweden nationwide, including ICUs from district, county and regional/university hospitals in both rural and urban areas of Sweden. The limitations include the lack of data regarding socioeconomic factors, smoking, and that we only have data for the first wave of the pandemic.

In conclusion, in this nationwide study of patients with COVID-19 in intensive care, we could demonstrate that a high BMI was associated with increased risk of death during intensive care and prolonged stay in the ICU. Importantly, the observed associations were not mediated via severity of illness at ICU admission. The results establish obesity as an independent risk factor for severe outcome from intensive care in patients with COVID-19. Based on our findings, we advocate that BMI is included in the severity scoring for patients with COVID-19 admitted to intensive care, and suggest that individuals with obesity should be more closely monitored when hospitalized for COVID-19.

## Supporting information

S1 Table Sex differences, age and comorbidities according to BMI categories. A cohort of 1,649 Swedish men and women treated at intensive care units during the first wave of the corona virus pandemic 2020. The data is presented as n(%).
(DOCX)

S2 Table BMI and BMI categories and the risk of composite outcome death or ICU stay over 14 days in individuals 55 years or less. Odds ratios (95% confidence intervals) for the composite outcome death during intensive care or length of stay (LOS) at intensive care unit over 14 days was calculated using logistic regression models. The adjusted model 1 is adjusted for age and sex, the adjusted model 2 is adjusted for age, sex and comorbidities. Reference group = ≥18, <25 kg/m^2^; Overweight = ≥25, <30 kg/m^2^; Obesity 1 = ≥30, <35 kg/m^2^; Obesity 2 and 3 = ≥35 kg/m^2^. BMI = body mass index, OR = odds ratio, CI = confidence interval.
(DOCX)

S3 Table BMI and BMI categories and outcomes for individuals *with COVID-19 as primary diagnosis*. Odds ratios (95% confidence intervals) for the composite outcome, death during intensive care or length of stay (LOS) at intensive care unit over 14 days in the population with COVID-19 as primary diagnosis (n = 1,552), was calculated using logistic regression models. Adjusted model 1 is adjusted for age and sex, adjusted model 2 is adjusted for age, sex and comorbidities, and adjusted model 3 is adjusted for age, sex, comorbidities and SAPS3. Reference group = ≥18, <25 kg/m2; Overweight = ≥25, <30 kg/m2; Obesity 1 = ≥30, <35 kg/m2; Obesity 2 and 3 = ≥35 kg/m2. Panel A: composite outcome; Panel B: death during stay at ICU, Panel C length of stay over 14 days. BMI = body mass index, OR = odds ratio, CI = confidence interval, LOS = length of stay, SAPS3 = acute physiology score 3.
(DOCX)

## References

[1] Bai Y, Yao L, Wei T, Tian F, Jin DY, Chen L, et al. Presumed Asymptomatic Carrier Transmission of COVID-19. Jama. 2020;323(14):1406–7. Epub 2020/02/23. doi: 10.1001/jama.2020.2565 ; PubMed Central PMCID: PMC7042844. 32083643 PMC7042844

[2] Gao Z, Xu Y, Sun C, Wang X, Guo Y, Qiu S, et al. A systematic review of asymptomatic infections with COVID-19. Journal of microbiology, immunology, and infection = Wei mian yu gan ran za zhi. 2021;54(1):12–6. Epub 2020/05/20. doi: 10.1016/j.jmii.2020.05.001 ; PubMed Central PMCID: PMC7227597. 32425996 PMC7227597

[3] Mizumoto K, Kagaya K, Zarebski A, Chowell G. Estimating the asymptomatic proportion of coronavirus disease 2019 (COVID-19) cases on board the Diamond Princess cruise ship, Yokohama, Japan, 2020. Euro surveillance: bulletin Europeen sur les maladies transmissibles = European communicable disease bulletin. 2020;25(10). Epub 2020/03/19. doi: 10.2807/1560-7917.Es.2020.25.10.2000180 ; PubMed Central PMCID: PMC7078829. 32183930 PMC7078829

[4] Treibel TA, Manisty C, Burton M, McKnight Á, Lambourne J, Augusto JB, et al. COVID-19: PCR screening of asymptomatic health-care workers at London hospital. Lancet (London, England). 2020;395(10237):1608–10. Epub 2020/05/14. doi: 10.1016/S0140-6736(20)31100-4 ; PubMed Central PMCID: PMC7206444. 32401714 PMC7206444

[5] Wu Z, McGoogan JM. Characteristics of and Important Lessons From the Coronavirus Disease 2019 (COVID-19) Outbreak in China: Summary of a Report of 72 314 Cases From the Chinese Center for Disease Control and Prevention. Jama. 2020;323(13):1239–42. Epub 2020/02/25. doi: 10.1001/jama.2020.2648 . 32091533

[6] Johansson MA, Quandelacy TM, Kada S, Prasad PV, Steele M, Brooks JT, et al. SARS-CoV-2 Transmission From People Without COVID-19 Symptoms. JAMA network open. 2021;4(1):e2035057. Epub 2021/01/08. doi: 10.1001/jamanetworkopen.2020.35057 ; PubMed Central PMCID: PMC7791354. 33410879 PMC7791354

[7] https://www.who.int/emergencies/diseases/novel-coronavirus-2019/situation-reports [accessed 20210630].

[8] www.socialstyrelsen.se/statistik-och-data/statistik/statistik-om-covid-19/statistik-over-antal-avlidna-i-covid-19/ [accessed 20210631].

[9] Barron E, Bakhai C, Kar P, Weaver A, Bradley D, Ismail H, et al. Associations of type 1 and type 2 diabetes with COVID-19-related mortality in England: a whole-population study. The lancet Diabetes & endocrinology. 2020;8(10):813–22. Epub 2020/08/18. doi: 10.1016/S2213-8587(20)30272-2 ; PubMed Central PMCID: PMC7426088. 32798472 PMC7426088

[10] Petrilli CM, Jones SA, Yang J, Rajagopalan H, O’Donnell L, Chernyak Y, et al. Factors associated with hospital admission and critical illness among 5279 people with coronavirus disease 2019 in New York City: prospective cohort study. BMJ (Clinical research ed). 2020;369:m1966. Epub 2020/05/24. doi: 10.1136/bmj.m1966 ; PubMed Central PMCID: PMC7243801 www.icmje.org/coi_disclosure.pdf and declare: support from the Kenneth C Griffin Charitable Fund for submitted work; no financial relationships with any organizations that might have an interest in the submitted work in the previous three years; no other relationships or activities that could appear to have influenced the submitted work. 32444366 PMC7243801

[11] Williamson EJ, Walker AJ, Bhaskaran K, Bacon S, Bates C, Morton CE, et al. Factors associated with COVID-19-related death using OpenSAFELY. Nature. 2020;584(7821):430–6. Epub 2020/07/09. doi: 10.1038/s41586-020-2521-4 . 32640463 PMC7611074

[12] Zhou F, Yu T, Du R, Fan G, Liu Y, Liu Z, et al. Clinical course and risk factors for mortality of adult inpatients with COVID-19 in Wuhan, China: a retrospective cohort study. Lancet (London, England). 2020;395(10229):1054–62. Epub 2020/03/15. doi: 10.1016/S0140-6736(20)30566-3 ; PubMed Central PMCID: PMC7270627. 32171076 PMC7270627

[13] Afshin A, Forouzanfar MH, Reitsma MB, Sur P, Estep K, Lee A, et al. Health Effects of Overweight and Obesity in 195 Countries over 25 Years. The New England journal of medicine. 2017;377(1):13–27. Epub 2017/06/13. doi: 10.1056/NEJMoa1614362 ; PubMed Central PMCID: PMC5477817. 28604169 PMC5477817

[14] Ball L, Serpa Neto A, Pelosi P. Obesity and survival in critically ill patients with acute respiratory distress syndrome: a paradox within the paradox. Critical care (London, England). 2017;21(1):114. Epub 2017/05/24. doi: 10.1186/s13054-017-1682-5 ; PubMed Central PMCID: PMC5440996. 28532465 PMC5440996

[15] Dai H, Alsalhe TA, Chalghaf N, Riccò M, Bragazzi NL, Wu J. The global burden of disease attributable to high body mass index in 195 countries and territories, 1990–2017: An analysis of the Global Burden of Disease Study. PLoS medicine. 2020;17(7):e1003198. Epub 2020/07/30. doi: 10.1371/journal.pmed.1003198 ; PubMed Central PMCID: PMC7386577. 32722671 PMC7386577

[16] Földi M, Farkas N, Kiss S, Zádori N, Váncsa S, Szakó L, et al. Obesity is a risk factor for developing critical condition in COVID-19 patients: A systematic review and meta-analysis. Obesity reviews: an official journal of the International Association for the Study of Obesity. 2020;21(10):e13095. Epub 2020/07/21. doi: 10.1111/obr.13095 ; PubMed Central PMCID: PMC7404429. 32686331 PMC7404429

[17] Huang Y, Lu Y, Huang YM, Wang M, Ling W, Sui Y, et al. Obesity in patients with COVID-19: a systematic review and meta-analysis. Metabolism: clinical and experimental. 2020;113:154378. Epub 2020/10/02. doi: 10.1016/j.metabol.2020.154378 ; PubMed Central PMCID: PMC7521361. 33002478 PMC7521361

[18] Simonnet A, Chetboun M, Poissy J, Raverdy V, Noulette J, Duhamel A, et al. High Prevalence of Obesity in Severe Acute Respiratory Syndrome Coronavirus-2 (SARS-CoV-2) Requiring Invasive Mechanical Ventilation. Obesity (Silver Spring, Md). 2020;28(7):1195–9. Epub 2020/04/10. doi: 10.1002/oby.22831 ; PubMed Central PMCID: PMC7262326. 32271993 PMC7262326

[19] Wu J, Li W, Shi X, Chen Z, Jiang B, Liu J, et al. Early antiviral treatment contributes to alleviate the severity and improve the prognosis of patients with novel coronavirus disease (COVID-19). Journal of internal medicine. 2020;288(1):128–38. Epub 2020/03/29. doi: 10.1111/joim.13063 . 32220033

[20] Gao M, Piernas C, Astbury NM, Hippisley-Cox J, O’Rahilly S, Aveyard P, et al. Associations between body-mass index and COVID-19 severity in 6·9 million people in England: a prospective, community-based, cohort study. The lancet Diabetes & endocrinology. 2021;9(6):350–9. Epub 2021/05/02. doi: 10.1016/S2213-8587(21)00089-9 ; PubMed Central PMCID: PMC8081400 diet replacement for weight loss funded by a grant from Cambridge Weight Plan UK to the University of Oxford (Oxford, UK) and received no personal payments from this. PA spoke at a symposium at the Royal College of General Practitioners annual conference on interventions for weight loss that was funded by Novo Nordisk and received no personal payments. JH-C reports grants from National Institute for Health Research (NIHR) Oxford Biomedical Research Centre (BRC), John Fell Oxford University Press Research Fund, Cancer Research UK (C5255/A18085) through the Cancer Research UK Oxford Centre, and Oxford Wellcome Institutional Strategic Support Fund (204826/Z/16/Z) during the conduct of the study. JH-C received personal fees and other support from ClinRisk (until 2019) outside of the submitted work, and is an unpaid director of QResearch, a not-for-profit organisation that is a partnership between the University of Oxford and Egton Medical Information Systems (EMIS) Health who supply the QResearch database used for this work. All other authors declare no competing interests. 33932335 PMC8081400

[21] Svensson P, Hofmann R, Habel H, Jernberg T, Nordberg P. Association between cardiometabolic disease and severe COVID-19: a nationwide case-control study of patients requiring invasive mechanical ventilation. BMJ Open. 2021;11(2):e044486. Epub 2021/02/19. doi: 10.1136/bmjopen-2020-044486 ; PubMed Central PMCID: PMC7893210. 33597145 PMC7893210

[22] Clinical characteristics and day-90 outcomes of 4244 critically ill adults with COVID-19: a prospective cohort study. Intensive care medicine. 2021;47(1):60–73. Epub 2020/11/20. doi: 10.1007/s00134-020-06294-x ; PubMed Central PMCID: PMC7674575. 33211135 PMC7674575

[23] Leong A, Cole J, Brenner LN, Meigs JB, Florez JC, Mercader JM. Cardiometabolic Risk Factors for COVID-19 Susceptibility and Severity: A Mendelian Randomization Analysis. medRxiv: the preprint server for health sciences. 2020. Epub 2020/09/11. doi: 10.1101/2020.08.26.20182709 ; PubMed Central PMCID: PMC7480065. 33661905 PMC7971850

[24] Díaz E, Rodríguez A, Martin-Loeches I, Lorente L, Del Mar Martín M, Pozo JC, et al. Impact of obesity in patients infected with 2009 influenza A(H1N1). Chest. 2011;139(2):382–6. Epub 2010/08/07. doi: 10.1378/chest.10-1160 . 20688928

[25] Fezeu L, Julia C, Henegar A, Bitu J, Hu FB, Grobbee DE, et al. Obesity is associated with higher risk of intensive care unit admission and death in influenza A (H1N1) patients: a systematic review and meta-analysis. Obesity reviews: an official journal of the International Association for the Study of Obesity. 2011;12(8):653–9. Epub 2011/04/05. doi: 10.1111/j.1467-789X.2011.00864.x . 21457180

[26] Huttunen R, Syrjänen J. Obesity and the risk and outcome of infection. International journal of obesity (2005). 2013;37(3):333–40. Epub 2012/05/02. doi: 10.1038/ijo.2012.62 . 22546772

[27] Kornum JB, Nørgaard M, Dethlefsen C, Due KM, Thomsen RW, Tjønneland A, et al. Obesity and risk of subsequent hospitalisation with pneumonia. The European respiratory journal. 2010;36(6):1330–6. Epub 2010/03/31. doi: 10.1183/09031936.00184209 . 20351023

[28] Moser JS, Galindo-Fraga A, Ortiz-Hernández AA, Gu W, Hunsberger S, Galán-Herrera JF, et al. Underweight, overweight, and obesity as independent risk factors for hospitalization in adults and children from influenza and other respiratory viruses. Influenza and other respiratory viruses. 2019;13(1):3–9. Epub 2018/12/06. doi: 10.1111/irv.12618 ; PubMed Central PMCID: PMC6304312. 30515985 PMC6304312

[29] Hunter A, Johnson L, Coustasse A. Reduction of intensive care unit length of stay: the case of early mobilization. The health care manager. 2014;33(2):128–35. Epub 2014/04/30. doi: 10.1097/HCM.0000000000000006 . 24776831

[30] Jolley SE, Bunnell AE, Hough CL. ICU-Acquired Weakness. Chest. 2016;150(5):1129–40. Epub 2016/04/12. doi: 10.1016/j.chest.2016.03.045 ; PubMed Central PMCID: PMC5103015. 27063347 PMC5103015

[31] Puthucheary ZA, Rawal J, McPhail M, Connolly B, Ratnayake G, Chan P, et al. Acute Skeletal Muscle Wasting in Critical Illness. Jama. 2013;310(15):1591–600. doi: 10.1001/jama.2013.278481 24108501

[32] https://www.cdc.gov/obesity/adult/defining.html

[33] Le Gall JR. The use of severity scores in the intensive care unit. Intensive care medicine. 2005;31(12):1618–23. Epub 2005/10/26. doi: 10.1007/s00134-005-2825-8 . 16244878

[34] Rees EM, Nightingale ES, Jafari Y, Waterlow NR, Clifford S, CA BP, et al. COVID-19 length of hospital stay: a systematic review and data synthesis. BMC medicine. 2020;18(1):270. Epub 2020/09/04. doi: 10.1186/s12916-020-01726-3 ; PubMed Central PMCID: PMC7467845. 32878619 PMC7467845

[35] https://www.folkhalsomyndigheten.se/folkhalsorapportering-statistik/tolkad-rapportering/folkhalsans-utveckling/resultat/halsa/overvikt-och-fetma/ [accesed 20210630].

[36] Laake JH, Buanes EA, Småstuen MC, Kvåle R, Olsen BF, Rustøen T, et al. Characteristics, management and survival of ICU patients with coronavirus disease-19 in Norway, March-June 2020. A prospective observational study. Acta anaesthesiologica Scandinavica. 2021;65(5):618–28. Epub 2021/01/28. doi: 10.1111/aas.13785 ; PubMed Central PMCID: PMC8014826. 33501998 PMC8014826

[37] Green WD, Beck MA. Obesity Impairs the Adaptive Immune Response to Influenza Virus. Annals of the American Thoracic Society. 2017;14(Supplement_5):S406–s9. Epub 2017/11/22. doi: 10.1513/AnnalsATS.201706-447AW ; PubMed Central PMCID: PMC5711276. 29161078 PMC5711276

[38] Chait A, den Hartigh LJ. Adipose Tissue Distribution, Inflammation and Its Metabolic Consequences, Including Diabetes and Cardiovascular Disease. Frontiers in cardiovascular medicine. 2020;7:22. Epub 2020/03/12. doi: 10.3389/fcvm.2020.00022 ; PubMed Central PMCID: PMC7052117. 32158768 PMC7052117

[39] Gong MN, Bajwa EK, Thompson BT, Christiani DC. Body mass index is associated with the development of acute respiratory distress syndrome. Thorax. 2010;65(1):44–50. Epub 2009/09/23. doi: 10.1136/thx.2009.117572 ; PubMed Central PMCID: PMC3090260. 19770169 PMC3090260

