# Supplementary material for: Impact of obesity on intensive care outcomes in patients with COVID-19 in Sweden—A cohort study
Source: PLoS One. 2021 Oct 13;16(10):e0257891. doi: 10.1371/journal.pone.0257891 (PMC8513867; doi:10.1371/journal.pone.0257891)
Supplement: S1 Table — A cohort of 1,649 Swedish men and women treated at intensive care units during the first wave of the corona virus pandemic 2020. The data is presented as n(%). (DOCX) [file pone.0257891.s001.docx]

**Supplemental material**

**Impact of obesity on intensive care outcomes in patients with COVID-19 in Sweden - a cohort study**

Lovisa Sjögren^1,2,3^, Erik Stenberg^4^, Meena Thuccani^5^, Jari Martikainen^6^, Christian Rylander^5^, Ville Wallenius^7^, Torsten Olbers^7^, Jenny M Kindblom^1,8^

### S1 Table Sex differences, age and comorbidities according to BMI categories

|  |  | **BMI categories** | | | | |
| --- | --- | --- | --- | --- | --- | --- |
|  | **Total** | **<18.0** | **≥18, <25** | **≥25, <30** | **≥30, <35** | **≥35** |
| **Cohort N(%)** | 1,649 | 15 (0.9) | 343 (20.8) | 641 (38.9) | 404 (24.5) | 246 (14.9) |
| **Females n(%)** | 422 | 9 (2.1) | 90 (21.3) | 124 (29.4) | 104 (24.6) | 95 (22.5) |
| **Males n(%)** | 1,227 | 6 (0.5) | 253 (20.6) | 517 (42.1) | 300 (24.4) | 151(12.3) |
| **Comorbidities, N(%)**  **0**  **1**  **2**  **3**  **4** | 1,034 (62.7)  258 (15.6)  222 (13.5)  129 (7.8)  6 (3.6) | 10 (66.7)  4 (26.7)  1 (6.7)  0 (0)  0 (0) | 218 (63.6)  63 (18.4)  42 (12.2)  18 (5.2)  2 (0.6) | 386 (60.2)  104 (16.2)  100 (15.6)  47 (7.3)  4 (0.6) | 259 (64.1)  57 (14.1)  47 (11.6)  41 (10.1)  0 (0) | 161 (65.4)  30 (12.2)  32 (13.0)  23 (9.3)  0 (0) |
| **Age, mean (SD)** | 60.6(13.0) | 67.3(13.9) | 63.1(13.1) | 61.6(12.4) | 58.1(12.1) | 54.6(13.6) |

A cohort of 1,649 Swedish men and women treated at intensive care units during the first wave of the corona virus pandemic 2020. The data is presented as n(%).
