# Supplementary material for: Impact of obesity on intensive care outcomes in patients with COVID-19 in Sweden—A cohort study
Source: PLoS One. 2021 Oct 13;16(10):e0257891. doi: 10.1371/journal.pone.0257891 (PMC8513867; doi:10.1371/journal.pone.0257891)
Supplement: S2 Table — Odds ratios (95% confidence intervals) for the composite outcome death during intensive care or length of stay (LOS) at intensive care unit over 14 days was calculated using logistic regression models. The adjusted model 1 is adjusted for age and sex, the adjusted model 2 is adjusted for age, sex and comorbidities. Reference group = ≥18, <25 kg/m2; Overweight = ≥25, <30 kg/m2; Obesity 1 = ≥30, <35 kg/m2; Obesity 2 and 3 = ≥35 kg/m2. BMI = body mass index, OR = odds ratio, CI = confidence interval. (DOCX) [file pone.0257891.s002.docx]

**Supplemental material**

**Impact of obesity on intensive care outcomes in patients with COVID-19 in Sweden - a cohort study**

Lovisa Sjögren^1,2,3^, Erik Stenberg^4^, Meena Thuccani^5^, Jari Martikainen^6^, Christian Rylander^5^, Ville Wallenius^7^, Torsten Olbers^7^, Jenny M Kindblom^1,8^

### S2 Table BMI and BMI categories and the risk of composite outcome death or ICU stay over 14 days in individuals 55 years or less

|  |  | **Unadjusted model** | **Adjusted model 1** | **Adjusted model 2** | **Adjusted model 3** |
| --- | --- | --- | --- | --- | --- |
|  | **N (cases)** | **OR per SD (95%SD)** | **OR per SD (95%SD)** | **OR per SD (95%SD)** | **OR per SD (95%SD)** |
| **BMI continuous** | 553 (231) | 1.31(1.13-1.52 | 1.40(1.19-1.64) | 1.47(1.24-1.73) | 1.47(1.24-1.75) |
| **BMI category** |  | **OR(95% CI)** | **OR(95% CI)** | **OR(95% CI)** | **OR(95% CI)** |
| **Reference group** | 86 (29) | Reference | Reference | Reference | Reference |
| **Overweight** | 174 (66) | 1.20(0.70-2.07) | 1.13(0.65-1.97) | 0.99(0.55-1.76) | 0.97(0.54-1.75) |
| **Obesity 1** | 169 (72) | 1.46(0.85-2.51) | 1.37(0.79-2.37) | 1.34(0.75-2.39) | 1.38(0.76-2.51) |
| **Obesity 2 and 3** | 122 (64) | 2.17(1.23-3.84) | 2.44(1.35-4.43) | 2.52(1.38-4.61) | 2.53(1.36-4.75) |

Odds ratios (95% confidence intervals) for the composite outcome death during intensive care or length of stay (LOS) at intensive care unit over 14 days was calculated using logistic regression models. The adjusted model 1 is adjusted for age and sex, the adjusted model 2 is adjusted for age, sex and comorbidities. Reference group = ≥18, <25 kg/m^2^; Overweight= ≥25, <30 kg/m^2^; Obesity 1= ≥30, <35 kg/m^2^; Obesity 2 and 3= ≥35 kg/m^2^.

BMI=body mass index, OR=odds ratio, CI=confidence interval
