# Supplementary material for: Impact of obesity on intensive care outcomes in patients with COVID-19 in Sweden—A cohort study
Source: PLoS One. 2021 Oct 13;16(10):e0257891. doi: 10.1371/journal.pone.0257891 (PMC8513867; doi:10.1371/journal.pone.0257891)
Supplement: S3 Table — Odds ratios (95% confidence intervals) for the composite outcome, death during intensive care or length of stay (LOS) at intensive care unit over 14 days in the population with COVID-19 as primary diagnosis (n = 1,552), was calculated using logistic regression models. Adjusted model 1 is adjusted for age and sex, adjusted model 2 is adjusted for age, sex and comorbidities, and adjusted model 3 is adjusted for age, sex, comorbidities and SAPS3. Reference group = ≥18, <25 kg/m2; Overweight = ≥25, <30 kg/m2; Obesity 1 = ≥30, <35 kg/m2; Obesity 2 and 3 = ≥35 kg/m2. Panel A: composite outcome; Panel B: death during stay at ICU, Panel C length of stay over 14 days. BMI = body mass index, OR = odds ratio, CI = confidence interval, LOS = length of stay, SAPS3 = acute physiology score 3. (DOCX) [file pone.0257891.s003.docx]

**Supplemental material**

**Impact of obesity on intensive care outcomes in patients with COVID-19 in Sweden - a cohort study**

Lovisa Sjögren^1,2,3^, Erik Stenberg^4^, Meena Thuccani^5^, Jari Martikainen^6^, Christian Rylander^5^, Ville Wallenius^7^, Torsten Olbers^7^, Jenny M Kindblom^1,8^

**S3 Table BMI and BMI categories and outcomes for individuals *with COVID-19 as primary diagnosis***

|  | **N (cases)** | **Unadjusted model** | **Adjusted model 1** | **Adjusted model 2** | **Adjusted model 3** |
| --- | --- | --- | --- | --- | --- |
|  |  | ***Panel A: Composite outcome*** | | | |
|  |  | **OR per SD (95%SD)** | **OR per SD (95%SD)** | **OR per SD (95%SD)** | **OR per SD (95%SD)** |
| **BMI continuous** | 1,552 (827) | 1.09(0.99-1.21) | 1.25(1.12-1.39) | 1.24(1.11-1.39) | 1.24(1.11-1.39) |
| **BMI category** |  | **OR(95% CI)** | **OR(95% CI)** | **OR(95% CI)** | **OR(95% CI)** |
| **Reference group** | 307 (162) | Reference | Reference | Reference | Reference |
| **Overweight** | 607 (314) | 0.96(0.73-1.26) | 0.98(0.74-1.30) | 0.95(0.71-1.26) | 0.98(0.74-1.31) |
| **Obesity 1** | 386 (206) | 1.02(0.76-1.38) | 1.23(0.90-1.69) | 1.18(0.86-1.64) | 1.25(0.89-1.73) |
| **Obesity 2 and 3** | 240 (140) | 1.25(0.89-1.76) | 1.70(1.16-2.48) | 1.62(1.10-2.37) | 1.64(1.12-2.42) |
|  |  | ***Panel B: death during intensive care*** | | | |
|  |  | **OR per SD (95%SD)** | **OR per SD (95%SD)** | **OR per SD (95%SD)** | **OR per SD (95%SD)** |
| **BMI continuous** | 1,552 (317) | 0.96(0.85-1.09) | 1.23(1.07-1.41) | 1.22(1.06-1.40) | 1.20(1.04-1.38) |
| **BMI category** |  | **OR(95% CI)** | **OR(95% CI)** | **OR(95% CI)** | **OR(95% CI)** |
| **Reference group** | 307 (65) | Reference | Reference | Reference | Reference |
| **Overweight** | 607 (124) | 0.96(0.68-1.34) | 1.06(0.74-1.50) | 1.05(0.84-1.66) | 1.14(0.79-1.64) |
| **Obesity 1** | 386 (79) | 0.96(0.66-1.39) | 1.50(1.00-2.25) | 1.48(0.98-2.22) | 1.53(1.02-2.32) |
| **Obesity 2 and 3** | 240 (45) | 0.86(0.56-1.31) | 1.42(0.89-2.29) | 1.37(0.85-2.21) | 1.39(0.86-2.26) |
|  |  | ***Panel C: LOS ≥14 days among survivors*** | | | |
|  |  | **OR per SD (95%SD)** | **OR per SD (95%SD)** | **OR per SD (95%SD)** | **OR per SD (95%SD)** |
| **BMI continuous** | 1,234 (509) | 1.14(1.02-1.29) | 1.22(1.08-1.38) | 1.23(1.09-1.39) | 1.23(1.09-1.39) |
| **BMI category** |  | **OR(95% CI)** | **OR(95% CI)** | **OR(95% CI)** | **OR(95% CI)** |
| **Reference group** | 242 (97) | Reference | Reference | Reference | Reference |
| **Overweight** | 482 (189) | 0.96(0.70-1.32) | 0.95(0.69-1.31) | 0.92(0.66-1.27) | 0.93(0.67-1.28) |
| **Obesity 1** | 307 (127) | 1.06(0.75-1.49) | 1.11(0.77-1.58) | 1.07(0.74-1.55) | 1.12(0.77-1.62) |
| **Obesity 2 and 3** | 195 (95) | 1.42(0.97-2.08) | 1.67(1.11-2.53) | 1.64(1.08-2.49) | 1.66(1.09-2.53) |

Odds ratios (95% confidence intervals) for the composite outcome, death during intensive care or length of stay (LOS) at intensive care unit over 14 days in the population with COVID-19 as primary diagnosis (n=1,552), was calculated using logistic regression models. Adjusted model 1 is adjusted for age and sex, adjusted model 2 is adjusted for age, sex and comorbidities, and adjusted model 3 is adjusted for age, sex, comorbidities and SAPS3. Reference group = ≥18, <25 kg/m2; Overweight= ≥25, <30 kg/m2; Obesity 1= ≥30, <35 kg/m2; Obesity 2 and 3= ≥35 kg/m2.

Panel A: composite outcome; Panel B: death during stay at ICU, Panel C length of stay over 14 days.

BMI=body mass index, OR=odds ratio, CI=confidence interval, LOS=length of stay, SAPS3=acute physiology score 3
